# The mechanism of whey protein and blueberry juice mixed system fermented with *Lactobacillus* inhibiting *Escherichia coli* during storage

**DOI:** 10.1038/s41598-023-33888-4

**Published:** 2023-04-24

**Authors:** Yu Qian, Lu Gui-bing, Wang Wen-qiong, Tang Cong-Cong, Gu Ruixia

**Affiliations:** 1 College of Food Science and Engineering, Yangzhou University, Yangzhou, 225127 Jiangsu China; 2 Weiwei Food and Beverage Co., LTD, Xuzhou, 221114 Jiangsu China; 3 Jiangsu Key Laboratory of Dairy Biotechnology and Safety Control, Yangzhou University, Yangzhou, Jiangsu China; 4 Shaanxi Key Laboratory of Environmental Engineering, School of Environmental and Municipal Engineering, Xi’an University of Architecture and Technology, Xi’an, 710055 China

**Keywords:** Behavioural methods, Biological models, Microbiology techniques

## Abstract

The aim of this study was to identify the antimicrobial effect and mechanism of whey protein and blueberry juice mixed systems fermented with *Lactobacillus* against *Escherichia coli* during storage. The whey protein and blueberry juice mixed systems were fermented with *L. casei* M54*, L. plantarum* 67,* S. thermophiles* 99 and *L. bulgaricus* 134 and had different antibacterial activities against *E. coli* during storage. The antimicrobial activity of the mixed whey protein and blueberry juice mixture systems was the highest, with an inhibition zone diameter of approximately 230 mm, compared with the whey protein or blueberry juice systems alone. There were no viable *E. coli* cells 7 h after treatment with of the whey protein and blueberry juice mixed systems as determined by survival curve analysis. Analysis of the inhibitory mechanism showed that the release of alkaline phosphatase, electrical conductivity, protein and pyruvic acid contents, and aspartic acid transaminase and alanine aminotransferase activity in *E. coli* increased. These results demonstrated that these mixed systems fermented with *Lactobacillus*, especially those containing blueberries, could inhibit the growth of *E. coli* and even cause cell death by destroying the cell membrane and cell wall.

## Introduction

*Escherichia coli (E. coli)*, which usually resides in the intestinal tracts of mammals, was previously regarded as nonpathogenic bacteria. Most *E. coli* strains in food are nonpathogenic to humans, but some unique strains of *E. coli,* such as Shiga toxin-producing *E. coli* O157:H7, are pathogenic to both humans and animals, especially infants and young animals, and can cause intestinal infections, including abdominal pain, bloody diarrhoea, haemorrhagic colitis, haemolytic uraemic syndrome and even death^1^. *E. coli* often attaches to raw and unpasteurized meat and dairy products causing food contamination and food-borne illnesses^2^. The reason why these bacteria are so difficult to remove is due to their ability to form biofilms. The biofilm matrix consists of proteins, polysaccharides and so on, and can protect the bacteria contained within the biofilm and allow the bacteria to adapt to environment^3^. In addition, another difficult problem is the emergence and spread of antibiotic-resistant bacterial strains^4^. Therefore, it is urgent that effective alternative approaches be developed to prevent and improve such chronic infections and diseases. In recent years, many studies have suggested that some natural ingredients in plants can inhibit the growth of various foodborne pathogens ^4^.

Blueberry have been recognized as one of the most valuable fruits that contain a large quantity of nutritious components including organic acids, vitamins, minerals and micronutrients^5^. Furthermore, blueberries are an excellent source of bioactive compounds, including anthocyanins, phenolic acids, and flavonols, which endow this fruit with a series of beneficial properties, such as cancer protection and antioxidant, anti-inflammatory, and antibacterial activities^6^. Previous studies indicated that the phytochemicals in lowbush wild blueberries inhibit the growth of *E. coli* O157:H7 and increased the membrane permeability of *E. coli* O157:H7^7^. In particular, the phenolics and anthocyanins in blueberries not only exhibit strong scavenging of free radicals such as ·OH and DPPH, but also inhibit the growth of *E. coli*, *Salmonella enteritidis*, *Staphylococcus aureus* and so on. The antimicrobial activity of these blueberry compounds may disrupt the integrity of the cell membranes of foodborne pathogens^8^. A study reported that probiotic-fermented blueberry juices improved the reducing power and showed stronger antibacterial activity against *E. coli* than fresh blueberry juice^9^. Another study noted that lactic acid bacteria have antibacterial effects due to the key antibacterial compounds they produce, such as bacteriocin, hydrogen peroxide and organic acids^10^. In addition, lactoferrin found in the whey protein fraction of milk can interact with the lipopolysaccharide layer of gram-negative bacteria, disrupt their outer membrane and enhance their susceptibility to the lysozyme by increasing membrane permeability^11^. However, the antibacterial effect and mechanism of a *Lactobacillus*-fermented whey protein and blueberry juice mixed system on foodborne pathogens is unclear. Thus, the inhibitory mechanisms of the mixed fermentation systems against foodborne pathogens during storage deserve deep investigation.

The objectives of this study were: (1) to examine the antibacterial effect of a *Lactobacillus*-fermented whey protein and blueberry juice mixed system on *E. coli* during storage, and (2) to investigate the possible antibacterial mechanism of this system from the perspective of cells membrane integrity. The *Lactobacillus*-fermented whey protein and blueberry juice mixed system showed high antibacterial activity (inhibition zone diameters and survival curves) against *E. coli*. Then, the cell membrane integrity (as determined by evaluating the activities of AKP, AST and ALT, electrical conductivity, and protein and pyruvate contents) was studied according to the inhibition mode. Therefore, the *Lactobacillus-*fermented whey protein and blueberry juice mixed system has potential for becoming a novel antibacterial agent and provides a basis for its application as an antibacterial agent in foods and medicines.

## Material and methods

### Materials

Whey protein (protein content 80%) was obtained from Fonterra group (Auckland, New Zealand). Blueberries (*Vaccinium angustifolium*) were purchased from farmers in Daxinganling, China. The bacterial strain *E. coli*, *Lacticaseibacillus casei* M54 (*L. casei* M54), *Lactiplantibacillus plantarum* 67 (*L. plantarum* 67), *Streptococcus thermophiles 99* (*S. thermophiles* 99) and *Lactobacillus bulgaricus* 134 *(L. bulgaricus* 134) were preserved in the Jiangsu Key Laboratory of Dairy Biotechnology and Safety Control, Yangzhou University. The kits were purchased from Nanjing Jiancheng Bioengineering Institute (Nanjing, China).

### Preparation of the blueberry and whey protein fermented beverages

The *Lactobacillus* strains were activated in 10 mL of Man-Rogosa-Sharpe (MRS) broth at 37 °C for 16 h. *S. thermophiles* was activated in 10 mL of M17 broth at 42 °C for 12 h. The mixed bacterial culture consisted of *L. casei* M54 and* L. plantarum* 67 at a ratio of 1:1, as well as *S. thermophiles* 99 and* L. bulgaricus* 134. The sample treatments of samples and *Lactobacillus* strains used were shown in Table 1. Blueberry juice was prepared from 17 g of blueberries by breaking the fruits with a crusher followed by filtering using a 200 mesh filter screen. Subsequently, the blueberry juice was mixed with distilled water to reach a blueberry juice content of 17% (w/v). One hundred millilitres of blueberry juice was added to 6 g (w/v) of sugar and 6 g (w/v) of whey protein powder. Then, the pH values of the blueberry and whey protein mixtures were adjusted to 6.5 and 7.0, and pasteurized in a water bath. After cooling to room temperature, 2 mL of the mixed bacterial culture was added to 100 mL of the blueberry and whey protein mixture to achieve an inoculum of 2% (v:v), respectively. The blueberry and whey protein mixtures fermented with* L. casei* M54 and* L. plantarum* 67 were incubated at 37 °C, and the other mixtures fermented with *S. thermophiles* 99 *and L. bulgaricus* 134 were incubated at 42 °C.

**Table 1:** The treatments of the samples.

Samples	pH	The strains used	
*Blueberry*	6.5	*L. casei* M54	*L. plantarum* 67
*L. casei* M54			
*L. plantarum* 67			
*Whey protein*	6.5	*L. casei* M54	*L. plantarum* 67
*L. casei* M54			
*L. plantarum* 67			
*Blueberry* + *Whey protein*	6.5	*L. casei* M54	*L. plantarum* 67
*L. casei* M54			
*L. plantarum* 67			
*Blueberry*	7.0	*S. thermophiles 99*	*L. bulgaricus* 134
*S. thermophiles 99*			
*L. bulgaricus* 134			
*Whey protein*	7.0	*S. thermophiles 99*	*L. bulgaricus* 134
*S. thermophiles 99*			
*L. bulgaricus* 134			
*Blueberry* + *Whey protein*	7.0	*S. thermophiles 99*	*L. bulgaricus* 134
*S. thermophiles 99*			
*L. bulgaricus* 134			

### Determination of the inhibition zone diameters

etermination of the *E. coli* inhibition zone diameters for the whey protein and blueberry juice mixed systems fermented with *Lactobacillus* was performed according to the method of Zhong et al.^9^ with slight modification. Briefly, 100 μL of 1 × 10^6^ CFU/mL *E. coli* suspensions was coated with glass coating rod uniformly on disposable sterilized solid agar plates. After 10 min, the medium was punched using a 10 mm diameter perforator that had been sterilized previously. Subsequently, 200 μL of the whey protein and blueberry juice mixtures fermented with *Lactobacillus* was placed into the holes with a pipette. Finally, the agar plates were placed in an electric 37 °C constant temperature incubator for 12 h.

### Survival curve analysis

Survival curves were used to determine the mode of antibacterial action of the whey protein and blueberry juice mixed systems fermented with *Lactobacillus* against *E. coli* according to the method of Zhou et al.^8^. In brief, all whey protein and blueberry juice mixed systems fermented with *Lactobacillus* were added to *E. coli* suspensions (10^9^ CFU/mL) to achieve a final concentration of 10^8^ CFU/mL. *E. coli* incubated with LB medium was regarded as the control sample. Subsequently, the above mixture were incubated at 37 °C for 12 h, and the reduction in the number of viable calls was measured every two hours by the plate counting method.

### Determination of the activity of AKP

The effects of the whey protein and blueberry juice mixed systems fermented with *Lactobacillus* on *E. coli* AKP activity were studied using an AKP assay kit according to the manufacturer's instructions. According to the method of Deng et al.^12^, the changes in absorbance at a wavelength of 520 nm represent the activity of AKP.

### Determination of the electrical conductivity

Determination of the electrical conductivity of the leaked bacterial cell contents was determined by measuring electrolyte leakage into the incubation medium with a conductivity meter (DDS-307, Precision & Scientific Instrument Co. Ltd., Shanghai, China). After incubation in LB medium at 37 °C for 12 h, *E. coli* were separated by centrifugation at 7000 r/min for 20 min, washed three times with triple 10 mM PBS (pH 7.0) and diluted with the same buffer to approximately 10^6^ CFU/mL. The samples fermented with *Lactobacillus* were then added to the bacterial cell suspensions. The mixtures were incubated with shaking (130 rpm) at 37 °C, and the conductivity was measured after 0, 4, 8 and 12 d^13^.

### Determination of the released protein content

The content of protein released from *E. coli* was determined using the BSA method. *E. coli* in the logarithmic phase were treated with the whey protein and blueberry juice mixed systems fermented with *Lactobacillus* for 10 h. The protein contents in the supernatants were assessed using the Pierce BCA Protein Kit (Thermo Scientific) purchased from Nanjing Jiancheng Bioengineering Institute (Nanjing, China)^14^.

### Determination of the activity of AST and ALT

The activities of AST and ALT were determined as described previously for the determination of AKP activity. The activities of AST and ALT in the supernatants were assessed using the AST and ALT Kit purchased from Nanjing Jiancheng Bioengineering Institute (Nanjing, China) according to the manufacturer's instructions^15^.

### Determination of the pyruvate content

The pyruvate content was determined from the measured AKP activity with some modifications. At first, the optical density of the supernatants at 600 nm was adjusted to 0.7. Then, the pyruvate content of *E. coli* was determined using a pyruvate kit purchased from Nanjing Jiancheng Bioengineering Institute (Nanjing, China) according to the manufacturer's instructions.

### Statistical analysis

Collected data are expressed as the means ± standard deviations (SDs). Analysis of variance (ANOVA) was performed, and the means were compared by the Student–Newman–Keuls test. A value of *p* < 0.05 was considered to indicate statistical significance. Data were analysed by using a statistical software package (SPSS for Windows, 11.5,2002, SPSS Inc., USA).

### Ethics approval and consent to participate

This experimental plant research (either cultivated or wild), including the collection of plant material, complied with institutional, national, or international guidelines. Field studies were conducted in accordance with local legislation. The plant materials have been widely consumed by local people and were used in our previous study.

### Ethical approval

This article does not contain any studies with human or animal subjects.

## Results and discussion

### Antimicrobial properties

**Figure 1:**
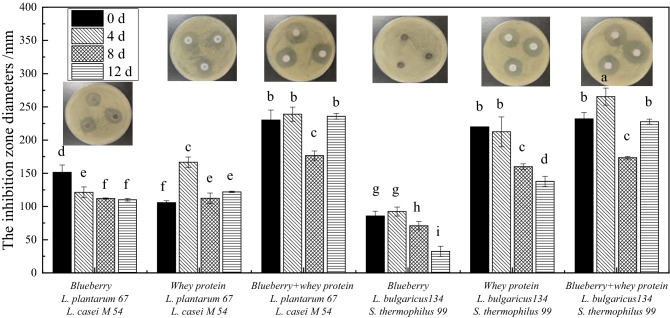
The inhibition zone diameters for whey protein and blueberry juice mixture system fermented with *Lactobacillus* against *E. coli.*

The antimicrobial properties of the whey protein and blueberry juice mixed systems fermented with *Lactobacillus* against *E. coli* during storage were determined by measuring the diameters of the zones of inhibition. As shown in Fig. 1, the antimicrobial activities of the blueberry and whey protein systems and the mixed systems fermented with *L. casei* M54 and* L. plantarum* 67 or *S. thermophiles* 99 and* L. bulgaricus* 134 were not significantly different. However, the blueberry and whey protein mixed fermentation systems showed the highest antimicrobial activity against *E. coli* after storage for 12 d, with inhibition zone diameters of approximately 230 mm and 225 mm, respectively. This result may be due to the increased release of polyphenols from the mixed blueberry and whey protein fermentation systems, as shown in our previous study^16^. It has been reported that polyphenols are the main antimicrobial agents affecting in vitro measurements^17^. Another report also suggested that the phenolics in blueberries are phytochemicals with strong antibacterial and antimycotic properties^9^. The antimicrobial activity of the blueberry and whey protein mixed system first increased and then decreased with prolonged storage time, which may be attributed to structural changes in the fermented antimicrobial products. In addition, the diameters of the antibacterial circles of the whey protein system fermented with *S. thermophiles* 99 and* L. bulgaricus* 134 against *E. coli* were 100 mm, 40 mm, 30 mm and 15 mm greater than those of the system fermented with *L. casei* M54 and* L. plantarum* 67, respectively. This effect may be closely related to the antimicrobial characteristics of lactic acid bacteria metabolites because different lactic acid bacteria have different metabolites including organic acids, fatty acids, short peptides and others^18^.

### Effects of the mixed systems fermented with *Lactobacillus* on the survival of *E. coli*

**Figure 2:**
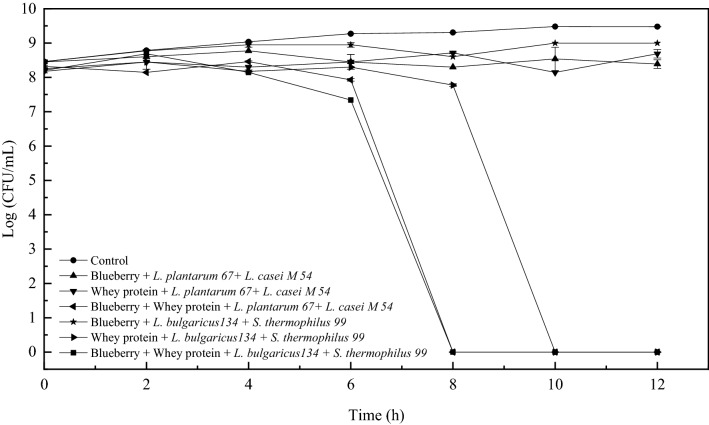
The survival curve of *E. coli* after the treatment of whey protein and blueberry juice mixture system fermented with *Lactobacillus.*

To gain an insight into the effects of the mixed systems fermented with *Lactobacillus* on the growth of *E. coli*, survival curve analysis was carried out. As depicted in Fig. 2, the untreated control *E. coli* cells grew steadily for 12 h to more than 9 log CFU in the initial stationary growth phase. Compared with the control, all systems fermented with *Lactobacillus* inhibited *E. coli* at the initial phase. With prolonged incubation time, the number of viable cells treated with the blueberry system increased. This result suggested that the blueberry system has bacteriostatic effects against *E. coli*^8^. In particular, the viable *E. coli* cell count after the bacteria were treated with the blueberry and whey protein mixed system fermented with *L. casei* M54 and* L. plantarum* 67, whey protein and the mixed system fermented with *S. thermophiles* 99 and* L. bulgaricus* 134 decreased from approximately 7 log CFU to 0 log CFU, which indicated that these fermentation systems have bacteriostatic and bactericidal effects on *E. coli*. The above results demonstrated these samples have the potential to become antibacterial agents against *E. coli*.

### Effect of the mixed fermentation systems on the activity of AKP

**Figure 3:**
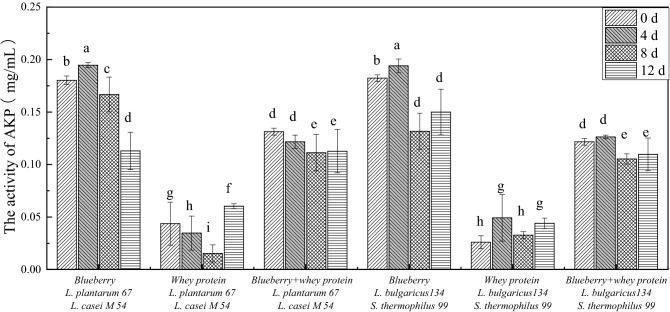
The activity of AKP treated by whey protein and blueberry juice mixture system fermented with *Lactobacillus.*

Cell membrane destruction has an unfavourable effect on the membrane-associated energy-transducing system. A study showed that the activity of AKP can regulate Ca^2+^ metabolism, which is related to calcium binding protein, the associated ion channels and energy metabolism based on phosphate. Once AKP is inactivated, cell differentiation is prevented by controlling the progress of dephosphorylation^4^. The effect of the blueberry and whey protein mixed system fermented with *Lactobacillus* on the AKP activity in *E. coli* was represented in Fig. 3. AKP activity of *E. coli* treated with blueberry system was higher than that of blueberry and whey protein mixed system and whey protein system. There was no significant difference between the systems fermented with *L. casei* M54, *L. plantarum* 67 and* S. thermophiles 99*, *L. bulgaricus* 134*.* Additionally, the activity of AKP was the highest when *E. coli* was treated with the blueberry system fermented with *L. casei* M54 and* L. plantarum* 67 (approximately 0.185 mg/ml) and the blueberry system fermented with *S. thermophiles* 99 and *L. bulgaricus* 134 (approximately 0.190 mg/ml) for 4 d. This result indicated that anthocyanins may enter the inner membrane of *E. coli* and stimulate AKP to flow out by disrupting the bacterial membrane and further inhibiting the growth of the bacteria^4^.

### Effects of the mixed fermentation systems on the electrical conductivity of the cultures

**Figure 4:**
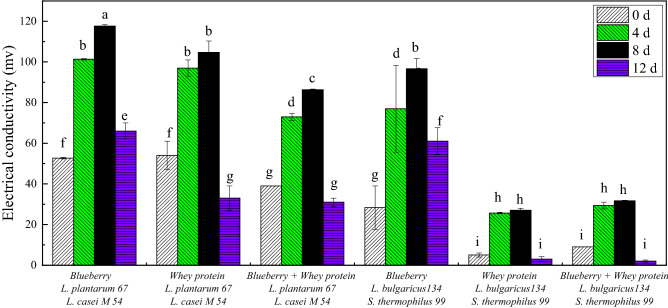
The electrical conductivity of *E. coli* cultures treated with whey protein and blueberry juice mixture system fermented with *Lactobacillus.*

The changes in the electrical conductivity of cell cultures treated with different samples were shown in Fig. 4, which were used to detect the permeability of the cell membrane. The electrical conductivity of *E. coli* cultures treated with all samples significantly (*p* < 0.05) increased during the first 4 days. Then, the electrical conductivity increased slowly. This result indicated that the *E. coli* cell membrane was disrupted and that the permeability of the cell membrane increased after treatment with all of whey protein and blueberry juice mixed systems fermented with *Lactobacillus*, which resulted in the leakage of intracellular ions such as Na^+^ and K^+^ that play a vital role in maintaining the balance of the organism and adjusting metabolic activity^19^. The results were in agreement with those that found an increase in relative electric conductivity of *E. coli* O157:H7 treated with different concentrations of natural antimicrobials for 2, 6 and 24 h^14^. A similar study suggested that treatment with *Rosa rugosa* tea polyphenol increased the permeability of the *S. aureus* cell membrane and caused ion leakage, which caused an increase in the electrical conductivity in the culture medium and finally cell death^20^. After storage for 12 days, the electrical conductivity of all treated *E. coli* decreased significantly (*p* < 0.05), which may be related to the reduction in phenolic compound contents in the samples. Previous study showed that the blueberry polyphenol content in fermented yogurt gradually decreased after storage for 28 days^21^. In particular, the electrical conductivities of the *E. coli* cultures treated with the systems fermented with *L. casei* M54 and *L. plantarum* 67 were relatively higher than those treated with *S. thermophiles* 99 and *L. bulgaricus* 134, which may be attributed to the production of different antibacterial substances. It was also suggested that the antibacterial substances of *L. plantarum* against *E. coli* are mainly organic acids and antimicrobial peptides^22^. The antibacterial effect of *L. bulgaricus* against *E. coli* has mainly been associated with lactic acid^23^.

### Effects of the mixed fermentation systems on the content of protein released from *E. coli*

Whether the cell membrane is intact affects the growth and survival of bacterial cells. The bacterial cell membrane is a selective permeability barrier to the internal and external environment that controls the penetration of nutrients to maintain normal bacterial growth and drain metabolic waste. Therefore, to investigate the cell membrane integrity of *E. coli* treated with the blueberry and whey protein mixed systems fermented with *Lactobacillus*, the content of cellular proteins leaked from *E. coli* was studied.

**Figure 5:**
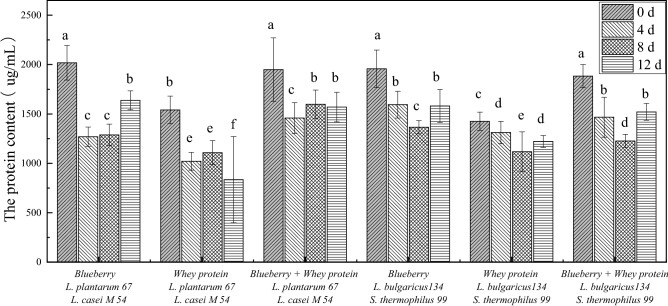
The released protein content of* E. coli* treated with whey protein and blueberry juice mixture system fermented with *Lactobacillus.*

Protein release from all samples remained stable, as shown in Fig. 5. The above results showed that the blueberry and whey protein mixed systems fermented with *Lactobacillus* have a significant inhibitory effect on *E. coli*. The content of leaked protein from the samples treated with the fermentation systems supplemented with blueberries were almost 33.33% higher than the contents leaked from the samples treated with the whey protein systems fermented with* L. casei* M54, *L. plantarum* 67 and *S. thermophiles* 99, *L. bulgaricus* 134 at 0 d. This result indicated that the main antibacterial compound may be associated with the components of blueberries. For example, the anthocyanin plus proanthocyanidin fraction plays a major role in damaging the integrity of the *E. coli* cell membrane^8^.

### Effects of the mixed fermentation systems on AST and ALT concentrations in *E. coli*

**Figure 6:**
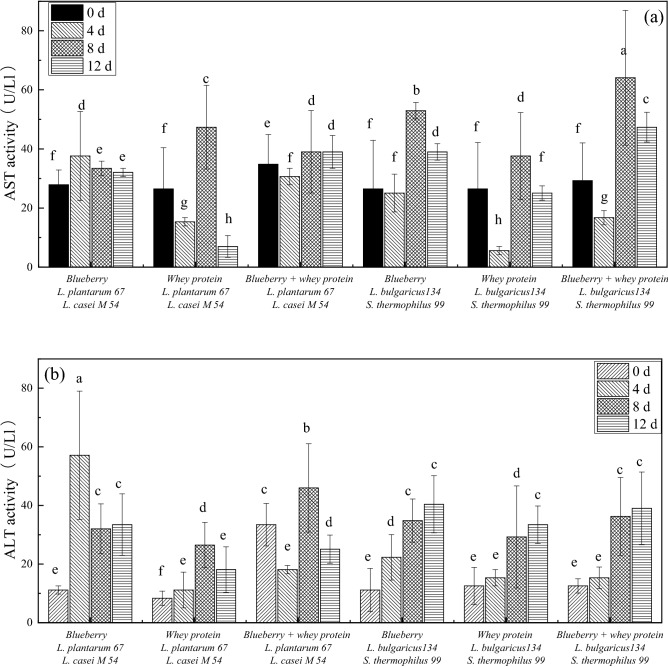
The AST (**a**) and ALT (**b**) activities in *E. coli* treated with whey protein and blueberry juice mixture system fermented with *Lactobacillus.*

AST and ALT are important intracellular enzymes in the metabolic pathways of organisms, participating in amino acid metabolism. With increasing cell wall and cell membrane permeability, intracellular enzymes can leak out. The concentrations of AST and ALT were measured to determine the integrality of cell wall and cell membrane, as shown in Fig. 6. The AST concentration was not significantly different among the blueberry systems, whey protein and blueberry systems. Nevertheless, the concentrations of AST in the bacterial cultures treated with the whey protein system were relatively lower during storage. Besides, the AST concentrations in *E. coli* treated with the whey protein system fermented with *L. casei* M54 and* L. plantarum* 67 were 20.83%, 142.86%, and 27.03% higher than those treated with the whey protein system fermented with *S.thermophiles* 99 and* L. bulgaricus* 134 after 8 days of storage. The ALT concentration results were roughly in agreement with those above. These results may be because antibacterial compounds such as bacteriocins that are produced by some *Lactobacillus* strains are more active at acidic pH than at neutral pH, which may have caused some of the antibacterial compounds to become inactive^24^.

### Effects of the mixed fermentation systems on the pyruvate content

**Figure 7:**
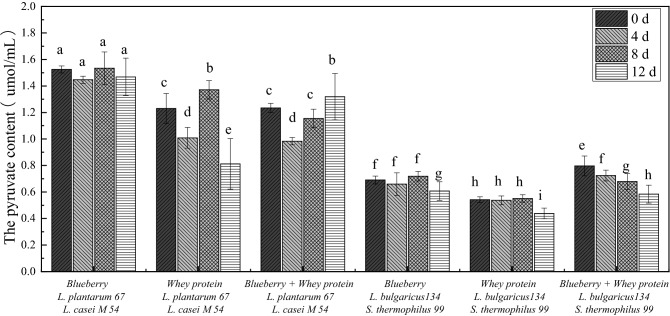
The pyruvate content in* E. coli* treated with whey protein and blueberry juice mixture system fermented with *Lactobacillus.*

Pyruvate plays an essential role in the TCA cycle (tricarboxylic acid cycle) and glycolysis process, participating in the energy metabolism pathways in living organisms^25^. Therefore, the pyruvate content was measured to determine whether the cell membrane retained its integrity. As shown in Fig. 7, the pyruvate contents in *E. coli* cultures treated with systems fermented with *L. casei* M54 and *L. plantarum* 67 were approximately 54%, 140% and 50% higher than those in the systems fermented with *S. thermophiles* 99 and* L. bulgaricus* 134 at 0 d, respectively. Nevertheless, there were no significant differences after 12 days of storage. The results showed that the mixed systems fermented with *L. casei* M54 and* L. plantarum* 67 can disrupt *E. coli* membrane integrity, increase the permeability of the cell membrane and cause the leakage of pyruvate, affecting bacterial energy metabolism and finally leading to cell death. This may be related to the antimicrobial properties of probiotics. The result may be connected with the fact that the organic acids and fatty acids metabolized by *L. plantarum*, such as lactic acid and acetic acid, have strong antibacterial activity and can destroy the cell membrane integrity of *E. coli* and result in the leakage of its cellular contents, finally affecting normal bacterial growth^18^.

## Conclusion

According to the above results, the fermentation systems that included blueberries were found to have the greatest antimicrobial effect against *E. coli*. For example, it was suggested that a whey protein and blueberry juice mixed system fermented with *Lactobacillus* displayed remarkable antibacterial characteristics against *E. coli* with antibacterial zone diameters of 230 nm and 225 mm at the end of storage, respectively. In particular, the blueberry systems had strong antibacterial effects against *E. coli*, as they disrupted the cell membrane integrity and affected the total content leakage, AKP activity, electrical conductivity and protein content. Additionally, the blueberry systems fermented with *L. casei* M54 and* L. plantarum* 67 also increased the pyruvate leakage, which interrupted normal growth of *E. coli* cells. Thus, the blueberry and whey protein mixed system fermented with *Lactobacillus* has great potential for future development as a natural antimicrobial agent to control pathogens. However, further studies are needed to explore what specific active components of the mixed systems fermented with *Lactobacillus* play a significant role in inhibiting the growth of *E. coli* and to investigate these antibacterial components against more microorganism strains. Furthermore, the results also indicated that the whey protein and blueberry juice mixed system fermented with *Lactobacillus* may become a promising natural preservative to prevent and control foodborne pathogens such as *E. coli*.

## Data Availability

The authors confirm that all data and materials as well as software applications to support their published claims and comply with field standards. All data can be provided upon request from the corresponding author.
